# Prior oral conditions in patients undergoing heart valve surgery

**DOI:** 10.4317/jced.53902

**Published:** 2017-11-01

**Authors:** Francisco-Javier Silvestre, Irene Gil-Raga, Mayte Martinez-Herrera, Dorina Lauritano, Javier Silvestre-Rangil

**Affiliations:** 1 Professor, Department od Stomatology, University of Valencia, Spain; 2 Diplomate of medical-surgery dentistry, University of Valencia. Spain; 3 Research fellow, Department of Stomatology, University of Valencia. Spain; 4 Professor of Medicine and Surgery, Department of Universitty of Milano-Bicocca. Italy; 5 Associate Professor, Department of Stomatology, University of Valencia

## Abstract

**Background:**

Patients scheduled for heart valve surgery should be free of any oral infectious disorders that might pose a risk in the postoperative period. Few studies have been made on the dental conditions of such patients prior to surgery. The present study describes the most frequent prior oral diseases in this population group.

**Material and Methods:**

A prospective, observational case-control study was designed involving 60 patients (30 with heart valve disease and 30 controls, with a mean age of 71 years in both groups). A dental exploration was carried out, with calculation of the DMFT (decayed, missing and filled teeth) index and recording of the periodontal parameters (plaque index, gingival bleeding index, periodontal pocket depth, and attachment loss). The oral mucosa was also examined, and panoramic X-rays were used to identify possible intrabony lesions.

**Results:**

Significant differences in bacterial plaque index were observed between the two groups (*p*<0.05), with higher scores in the patients with valve disease. Probing depth and the presence of moderate pockets were also greater in the patients with valve disease than among the controls (*p*<0.01). Sixty percent of the patients with valve disease presented periodontitis.

**Conclusions:**

Patients scheduled for heart valve surgery should be examined for possible active periodontitis before the operation. Those individuals found to have periodontal disease should receive adequate periodontal treatment before heart surgery.

** Key words:**Valve disease, aortic, mitral, heart surgery, periodontitis.

## Introduction

Heart valve diseases are an important cause of morbidity among elderly people all over the world. In the concrete case of Spain, where the population life expectancy is very long, such diseases become particularly important. Heart valve disorders comprise a series of congenital and acquired conditions, the underlying causes of which have varied over the last decades (1).

All of the heart valves can suffer alterations due to different etiologies such as myxomatous mitral valve disease or senile calcified aortic valve stenosis (2). Less frequent presentations include severe tricuspid valve regurgitation secondary to pacemaker implantation (3). Echocardiography is an important tool that allows the identification of anatomical heart valve changes in patients without a prior diagnosis (4). In this context it is important to consider optimum timing of treatment, without waiting for the symptoms to become serious (5).

Dental infections, and particularly periodontal disease, have been associated to cardiovascular disorders (6,7), though clinical confirmation in such cases is complicated by the influence of common risk factors and confounding factors (8-10). In the preoperative period of cardiovascular surgery, patients should be screened for the possible existence of odontogenic infections caused by dental caries or periodontitis, and which can result in increased morbidity-mortality (11,12). In this regard, patients that have not received preoperative dental treatment might be at increased risk in the postoperative period.

Few studies have been published on the presurgical oral health of patients scheduled for heart valve surgery. Such individuals have been found to have few remaining teeth, with important dental caries and periodontal alterations, though apart from the typically advanced age of such patients, the possible causal factors underlying these alterations have not been established (11-13).

The present study was carried out to identify the most frequent oral disorders in patients during the preoperative period of heart valve surgery.

## Material and Methods

-Study design and population

A prospective, case-control study was designed to determine the oral condition of patients scheduled for heart valve surgery and referred from the Department of Cardiology to the Stomatology Unit of Dr. Peset University Hospital (Valencia, Spain).

The study was approved by the Clinical Research Ethics Committee of the University of Valencia, and written informed consent was obtained from each patient.

We initially selected 33 individuals, though three failed to meet the inclusion criteria and were thus excluded from the study, leaving a final total of 30 patients with a mean age of 71.5 ± 9.5 years (21 females and 9 males). The included patients were required to be scheduled for heart valve surgery; have no serious systemic disorders other than heart valve disease; and have at least 10 teeth in the mouth.

Sample size calculation was carried out to establish case-control comparisons of the quantitative effect variables (plaque index, bleeding index, periodontal pocket depth, etc.) with a statistical power of 80% and an alpha error of 0.05, capable of detecting a standardized difference of 0.8 according to the Cohen scale (14). Sample Power 2.0 (SPSS Inc., Chicago, IL, USA) was used to establish a total of 26 patients per group. We raised this figure slightly to 33 individuals per group, with the final inclusion of 30, in prevision of possible missing data.

An age- and gender-matched control group (mean age 71.2 ± 9.2 years; 21 females and 9 males) was recruited on a randomized basis from among the people accompanying the patients. Apart from the absence of heart disease (and therefore no need for cardiovascular surgery), the controls were required to meet the same inclusion criteria as the patients.

-Study parameters

The case history was compiled, with the collection of information on general health and a thorough oral examination, with the obtainment of panoramic X-rays in all subjects. The DMFT (decayed, missing and filled teeth) index was recorded with the help of an intraoral dental mirror and probe. All the teeth were assessed for active caries and root fragments; fillings, crowns and bridges; and endodontic treatment. Missing teeth were evaluated clinically and radiologically. The sum of the components of the DMFT scale was divided by the number of teeth in the mouth.

We also recorded periodontal parameters such as plaque index (PI), bleeding index (BI), pocket depth (PD) and clinical attachment loss (CAL). All the mesial and palatine or lingual surfaces of the teeth were evaluated in order to calculate the plaque index, scored from 0-4 (0 = not detectable; 1 = only detectable with the probe; 2 = moderate and visible; 3 = abundant plaque extending beyond the cervical third of the tooth). The mean score of all the teeth was calculated.

Pocket depth in turn was measured at 6 points per tooth (3 buccal and 3 palatine or lingual): a depth of up to 3 mm was considered physiological; 4-5 mm corresponded to moderate pockets; and ≥ 6 mm was taken to be indicative of severe periodontal pockets.

The bleeding index was measured by probing at four points, calculating the mean bleeding area per explored surface.

Recession was calculated as the distance (in mm) between the cementoenamel junction and the free gingival margin, while clinical attachment loss was determined as the distance (in mm) between the cementoenamel junction and the depth of the periodontal pouch.

The oral mucosa was also thoroughly explored in order to detect possible structural changes or anomalies: tongue, lips, check mucosa, gums, palate (hard and soft), floor of the mouth, oropharyngeal isthmus and epiglottis.

Lastly, the panoramic X-rays images were evaluated for possible radiopacities, translucencies or mixed images.

-Statistical analysis

A descriptive analysis was made of the study variables, reporting continuous variables as the mean and standard deviation, and categorical variables as frequency and percentage. The Student t-test for dependent samples was used to establish comparisons between the case and control groups. Normal data distribution was previously confirmed using the Kolmogorov-Smirnov test (14). The chi-squared test in turn was used to establish associations between categorical variables.

Due to the limited sample size, the Mann-Whitney U-test was used to contrast parameters according to the type of valve disease involved (mitral or aortic). Statistical significance was considered for *p* ≤ 0.05.

## Results

Both groups (case and control) involved the same number of individuals (n=30) and were homogeneous in terms of age and gender distribution ( Table 1).

**Table 1:**
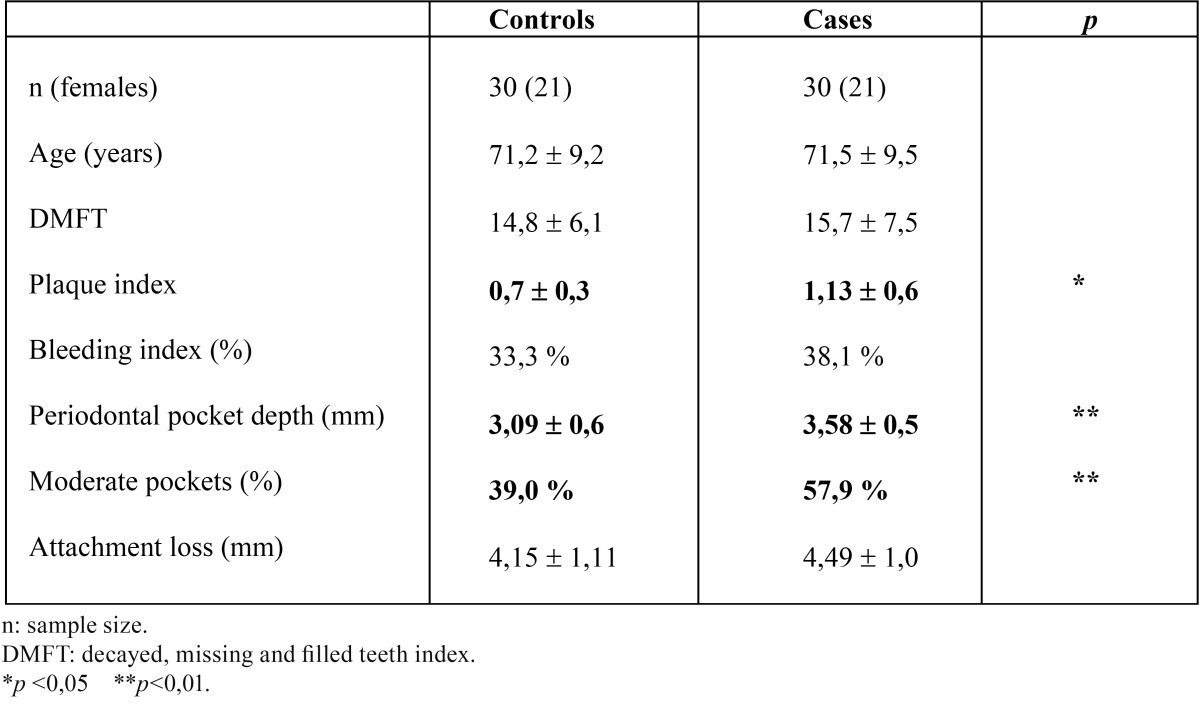
Simple size, age, main caries and periodontal parameters measured between the group of patients undergoing heart valve surgery and control group.

The mean number of teeth in the mouth was 20.2±5.2 among the patients and 20.2±6.0 in the control group.

The mean DMFT index was 15.7±7.5 in the patients scheduled for heart valve surgery and 14.8v6.1 in the controls – the difference between groups being no significant (*p*=0.594).

The mean dental plaque index was significantly higher in the patient group (1.13±0.62 versus 0.76±0.39; *p*=0.023).

The bleeding index tended to be greater in the patients with valve disease (38.10% versus 33.31% in the controls), though significance was not reached (Student t-test).

The mean pocket depth was significantly greater among the patients (3.58±0.53 versus 3.09±0.69 mm in the control group; *p*=0.004). However, on considering the different depth intervals (physiological, moderate, severe), statistical significance was seen to be limited to moderate pocket depth (57.9±25.4% in the patients versus 39.0±15.7% in the controls; *p*=0.002).

Gingival recession was greater among the controls than in the patients with valve disease (1.07 mm versus 0.91 mm; *p*=0,552), though attachment loss was greater among the latter (4.49±1.01mm versus 4.15v1.11 mm in the control group; *p*=0.265).

Most of the patients with heart valve disease had no oral mucosal lesions; only two small angiomas and one fibroma of the cheek mucosa were diagnosed. Likewise, the panoramic X-rays revealed no bone lesions.

On dividing the patients according to the type of valve disorder, 16 were seen to suffer aortic valve stenosis (8 females and 8 males, with a mean age of 71.5±9.5 years), while 14 presented mitral valve stenosis (13 females and one male, with a mean age of 73.1±6.9 years).

The number of teeth present in the mouth was similar in both subgroups (19.9±5.8 in the patients with aortic valve stenosis versus 20.6±6.4 in those with mitral valve stenosis). Lastly, the DMFT score was 17.3±8.2 in the aortic stenosis subgroup and 14.0±6.5 in the mitral valve stenosis subgroup (*p*=0.21).

There were no differences between the two groups in terms of oral mucosal lesions or the panoramic X-rays findings.

## Discussion

Oral infections may pose a risk during the postoperative period of heart valve surgery (15). It has been seen that patients scheduled for cardiovascular surgery have poorer oral hygiene than the general population. The present pilot study was therefore designed to evaluate the presence of periodontal and oral infectious disease in patients before heart valve surgery. No studies of this kind have been carried out to date in Spain, and only limited information is available referred to populations in other countries such as Sweden, North America and Japan (11,12,16).

Our results show oral health in the patients scheduled for heart valve surgery to be slightly poorer than in individuals without valve disease. In effect, the dental plaque scores were higher, reflecting poorer dental hygiene among the cases versus the controls, and this may imply a greater risk of periodontal disease. One-half of the patients scheduled for heart valve surgery were seen to have moderate active periodontitis.

With regard to the homogeneity of the two study groups, the number of remaining teeth in the mouth and smoking habit were very similar in both cases. A direct relationship therefore may be postulated between increased plaque and the presence of a greater number of pockets of moderate depth, as well as greater attachment loss.

One of the greatest differences between groups was referred to dental plaque. We have found no studies specifically describing the amount of dental plaque in patients with heart valve disease. In contrast, the literature contains some studies on the periodontal health of such patients. Terezhalmy *et al.* (15) found 43.6% of their sample to have periodontitis, in coincidence with the observations of Nakamura *et al.* (12), who found almost one-half of the patients with valve disease to have advanced periodontitis. Another study evidenced alveolar bone loss of up to 70% in patients of this kind (11). These studies did not address the cause of such an increased prevalence of periodontitis, however.

In our series, almost 60% of the patients scheduled for heart valve surgery suffered periodontitis. This situation would be a consequence of plaque accumulation secondary to poorer oral hygiene, and could favor the appearance of bacteremia following tooth brushing in these individuals (17). Although periodontal bacteria have been isolated from heart valve tissues of patients with periodontitis (18), heart valve colonization by periodontal pathogens does not seem to be as frequent as once believed, in view of the bacterial adhesion and colonization difficulties posed by the high blood flow pressure levels reached in such areas (19,20).

Bacteremia secondary to periodontal infection is known to be one of the primary causes of infectious endocarditis (21). In particular, patients with heart valve disorders are at an increased risk. The American Heart Association therefore recommended dental exploration and the treatment of buccodental disease prior to performing heart surgery (22). Periodontal treatment is advised in patients with advanced periodontitis, followed by root scaling and ultrasound treatment. Those teeth not amenable to treatment and with a poor prognosis should be removed (12).

Aortic and mitral valve degeneration and calcification are known to be related to advanced age, and are characterized by risk factors similar to those found in arteriosclerosis. The possible association between arteriosclerosis - ischemic heart disease and chronic periodontitis has been reported in the literature (23,24). In contrast, the evidence relating periodontitis to heart valve disease has been more limited.

In our series moderate periodontitis was seen to be more prevalent, while the literature describes a greater presence of severe periodontitis. However, previous studies have not been able to demonstrate that dental treatment before heart valve surgery effectively improves survival over the short or middle term (25,26). On the other hand, some authors suggest that there are differences in the oral disease conditions found in patients with aortic valve disease versus those with mitral valve disorders (27). No such differences were observed in our series, since both subgroups (aortic and mitral valve disease) presented similar findings in terms of oral disease and levels of periodontitis.

In conclusion, we consider that patients scheduled for heart valve surgery should receive treatment for periodontitis, where required, avoiding plaque accumulation and improving oral hygiene and thus periodontal health. Such presurgical preventive measures are moreover inexpensive and accessible.
